# Observations on the Treatment of the Malignant Yellow Fever, Which Prevailed Partially in the City and Liberties of Philadelphia, in the Summer and Autumn of 1802

**Published:** 1803-02-01

**Authors:** William Currie

**Affiliations:** Fellow of the Colledge of Physicians, &c.


THE
Medical and Phyfical Journal.

VOL. IX.] FEBRUARY 1, 1803. [NO. XLVIII.

Printed for R. PHILLIPS, by W. Thorne, Red Lion Court, Fleet Street, London.

*Obfervations on the Treatment of the* MALIGNANT YELLOW FEVER, *which prevailed partially in the City and Liberties of Philadelphia, in the Summer and Autumn of* 1802. *Communicated by* WILLIAM CURRIE, *Fellow of the Colledge of Phyficians, &c.*

THE malignant Yellow Fever invaded the city of Philadelphia again this year, but its progress was more limited than heretofore. We have the moft incontestible proofs that it was introduced into Philadelphia this year by a ship, called the St. Domingo Packet, which arrived at our Lazaretto in June, and came up to the city, and moored at a wharf at its northern extremity on the 28th of June. Two persons had died on board this vessel at Cape François, and two after her arrival in the river Delaware. Her cargo had been landed, and the bedding of the seamen had been purified; but no attempt had been made to irradicate infection from the cabin or forecastle, places equally, if not more liable to be contaminated by the effluvia of the sick than any other parts of the vessel. The disease was originally confined to a very small number of persons, and the first persons attacked had been on board, or employed very near, the St. Domingo Packet, and all that were afterwards attacked, for the first two weeks, have been traced to intercourse with some of the sick. Its progress was slow, and similar to that of the putrid sore throat and other diseases unquestionably contagious, and no case had been taken, in any part of the city, previous to the arrival of that vessel.

The Board of Health warned the citizens of their danger early, in consequence of which three-fourths of the inhabitants removed, from the neighbourhood where the disease first appeared, into the country, by which means it was for a time nearly arrested; but some persons who had visited the sick, had unfortunately conveyed the infection to other neighbourhoods before that salutary measure was adopted; in consequence of which new cases again occurred, and gradually increased. But owing (in addition to the early desertion of the inhabitants) to the coolness of the air, in consequence of frequent thunder, and extraordinary floods of rain, the number taken sick from the 4th of July, when the first cases occurred, to the 1st of November, when the disease (after the occurrence of a few cold nights which produced ice) entirely ceased, only amounted to 584; but it is with regret I have to relate that of these 584, two hundred and ninety-eight died, including fifty-eight that died at the hospital, into which one hundred and ten had been admitted from the 1st of August to the 1st of November inclusive. The greatest mortality, in proportion to the number sick, occurred between the 17th and 25th of October, for of about sixty persons attacked with the fever within that period, near forty died. The disease also made its appearance, during that period, in a greater number of places detached from one another, than any other time this season. A black frost, in the night of October 26, gave a sensible check to the progress of the disease, after which it rapidly declined, and entirely ceased before the middle of November. From this statement, it is evident that the disease has been as mortal this year, in proportion to numbers, as at any former period, the year 1798 excepted, notwithstanding the tremendous thunder which frequently agitated the atmosphere, and the floods of rain by which the city, as well as the whole country, was repeatedly drenched during the hot season, and the consequent coolness of the air and freedom of ventilation which prevailed the remainder of the season.

Mercury was generally employed, both internally and externally, for the purpose of exciting salivation as speedily as possible, both at the hospital and in private practice; but, if I can trust my observations, seldom with success, excepting when employed at the very commencement of the disease, and so conducted as to affect the mouth before the dangerous symptoms of the second stage had time to make their appearance.

When employed in the second stage of the disease, at which time the predominant symptoms are generally disordered stomach, restlessness, oppression, and deep sighing, and a countenance that denotes great misery; it constantly aggravated the disease, and hurried on the fatal symptom of *black vomiting*.

In this stage of the disease, when the recited symptoms predominated, the frequent exhibition of mild laxatives in in small doses, particularly Rochell salts, soda phosphorata, soluble tartar, castor oil, senna and cream of tartar; and when these could not be retained, laxatives and clysters, were the most successful remedies, especially when aided by the application of blisters to the stomach, wrists and ancles, at the same time.

A solution of carbonat of soda in water, which is much more palatable than the vegetable alkali, followed immediately by a table spoonful of diluted lemon juice, or cream of tartar in water, had also sometimes the effect of allaying the distressing propensity to puke. But these as well as every other means that I have seen tried, too frequently failed of affording relief.

As dissections shew that the constant propensity to puke, which so generally occurs on the third day in this disease, is owing to an inflammation of the surface of the stomach, and as the indication is to remove this inflammation, what effect would iced water or iced cream have upon it?

Cupping upon the stomach, or drawing blood by the application of leeches, and blistering the stomach, are certainly indicated; but I have had no experienee of the effects of cupping, or of drawing blood by leeches in this disease.

In one case, the disorder of the stomach ceased immediately after the application of a large poultice of mustard flour to the stomach and feet, which occasioned a very extensive and painful inflammation of the skin.

In another case, lime-water diluted with simple cold water, given in small and repeated draughts, had a similar effect.

I have a favourable opinion of the warm bath in the second stage of this disease, especially when blisters or sinapisms are employed immediately after its application, by which means the circulation is more powerfully determined to the surface; but I had no opportunity of employing it this season for want of suitable assistance: for, rendered cruel and inhuman by the dread of contagion,

" Dependants, friends, relations, love himself,
" Forgot the tender tie,
" The sweet endearments of the feeling heart."

Though stimulating remedies and an invigorating regimen appeared indicated in the second stage of this fever, the inflamed and irritable state of the stomach generally rendered them inadmissable and highly dangerous.

 In

In this state of the stomach, the internal use of mercury, either alone, or when combined with opium, always increased the distressing propensity to puke; and when it failed to operate by stool, it aggravated every symptom of the disease; and the internal application of mercury, except when begun with at an earlier period, seldom produced any sensible effect. Dr. Davidson, of St. Lucia, in a letter to Dr. Buxton, dated October 14th, 1801, recommends for the relief of the distressing vomiting which so generally attends the second stage of this disease, a clyster of assafœtida with the addition of *two hundred drops* of liquid laudanum, and afterwards the extract or tincture of opium to be given by the mouth at proper intervals to prevent the return of this dangerous symptom; and he asserts that although he has seen this complaint aggravated by opiates given by the mouth, they have always afforded relief when given in clysters.

Whether, under these circumstances, laudanum given as recommended by Dr. Davidson, is entitled to the credit he ascribes to it, my experience does not qualify me to determine; but I have employed it several times in the quantity of sixty or eighty drops in a warm clyster, made of mucilaginous and aromatic vegetables infused in boiling water, in cases where vomiting, oppression about the precordia, and great irritability appeared to be owing to exhaustion from too copious depletion, with the most immediate and sensible benefit.

In cases of black vomiting, I am greatly deceived if I have not witnessed laudanum of great service, more than once given in a clyster made of a strong decoction of bark and serpentaria, frequently repeated.

This dreadful symptom, however, has been more frequently relieved by giving from one to four table spoons full of an equal quantity of lime water and new milk mixed together, every hour or oftener, than by any other remedy, when employed on the first appearance of that symptom.

In the year 1798, in the case of Mr. Fendal, it completely relieved him of that distressing and ominous symptom, and he recovered after he had been reduced to the most hopeless extremity.

Several instances are mentioned by Dr. Hossack, (who first brought lime-water into credit) of its success at New York; and Dr. Vaughan, of Wilmington, informs me, that a person recovered under his care by the use of it this season

Perhaps

Perhaps this remedy, from its sedative and mildly astringent quality, as well as from its correcting the acid which generally abounds in the stomach previous to the commencement of the black vomiting, would be more efficacious in preventing than in removing this symptom, by preventing the sphacelated condition of the surface of the stomach, to which it is owing, if employed earlier in the disease.

In one case this autumn under my care, given diluted with water, it settled the stomach, when laxatives and clysters were of no avail. In another case, carbonat of soda, in doses of 20 grains, dissolved in water, and frequently repeated, had the same effect.

In cases where the disease began with strong action of the arteries, severe pain in the head, back, and limbs, with little or no sickness at stomach; bleeding, purging with active medicines, and the strict observance of every part of the antiphlogistic regimen, generally occasioned a partial solution of the fever on the third, and a complete solution on the fifth day from the attack. In these cases, wetting the patient's head, face, and limbs frequently with cold water, had a sedative and salutary effect; but neither the plunging nor shower-bath was employed this year.

This form of the disease resembles the eruptive fever of the distinct small-pox, and is generally subdued by the same remedies as render the pustules few and the subsequent symptoms mild. When, however, these were omitted, or employed too sparingly, the remission of the fever was frequently followed by the different and more dangerous train of symptoms peculiar to the second stage of the disease.

When the disease began with weak pulse, general debility, great oppression at the precordia, constant restlessness, and deep and frequent sighing, I have thought myself justifiable, from the analogy of the disease with the putrid sore throat (in which mercury has been found more successful in this country than any other remedy) as well as from the testimony of Dr. Chisholm, (who first employed it for the purpose of exciting salivation in this disease) in favour of its safety and superior efficacy, to have recourse to mercury, and have given calomel in doses of 4 grains every three hours, day and night, carefully guarding it from passing off by stool, by combining it with a sufficient quantity of opium till the mouth became affected by it; and have made it a rule always to begin with it,

 before before the symptoms of the second stage commenced, or not at all.

Confiding in the observations of Dr. Patterson, a surgeon in the British army, who declares, in a letter to Dr. Chisholm, that he has found the early application of blisters to the stomach and limbs prevent a determination to the stomach, I have in every case where I prescribed mercury, directed a large blister to the stomach, wrists, and ancles at the same time, and as early as possible in the disease. And after the removal of the blisters, have dressed the excoriated surface every four or five hours with strong mercurial ointment, and have also directed it to be rubbed liberally upon different parts of the patient's body at the same time, when insuperable prejudices were not opposed to its use, which unfortunately was too often the case, owing to the ill effects of its indiscriminate and improper application in the late stages of the disease.

Though my success with this mode of treatment was not very flattering, it certainly failed less frequently than any other that I have hitherto witnessed; and if I could have employed the warm bath as an auxiliary I have reason to believe it would have been much more successful.

In every case, however where I have seen mercury employed after the distressing and dangerous symptoms of the second stage had come on, it always aggravated the symptoms and increased the danger; and when employed after signs of what is called putrescency or scurvy have made their appearance, such as purple spots, extravasations under the cuticle, and hæmorrhagies from different parts of the body, the exhibition of mercury has invariably accelerated the fatal event, notwithstanding the declaration of Dr. Chisholm to the contrary.

Though salivation may generally be considered as a pledge of security in this formidable disease, a few instances have come under my notice of its failure after salivation had plentifully taken place. These, however, have been so rare, that I am inclined to think it the duty of every Physician acquainted with this circumstance, to make the accomplishment of it the principal object of his aim. I am also persuaded, that the difficulty of exciting salivation is much owing to the lateness of the application, and the omission of suitable auxilliaries.

No restrictions are necessary with regard to the application of mercury, externally, at any period of the disease, so long as the extremities continue warm, and the absorbents preserve their power; but the internal exhibition of it, it, at any period after the stomach has become constantly disordered, is not only improper but in the highest degree dangerous and destructive.

*Philadelphia, Nov. 20, 1802.*

---

